# Tipping the balance: toward rational combination therapies to overcome venetoclax resistance in mantle cell lymphoma

**DOI:** 10.1038/s41375-022-01627-9

**Published:** 2022-06-20

**Authors:** Yvonne J. Thus, Eric Eldering, Arnon P. Kater, Marcel Spaargaren

**Affiliations:** 1 Department of Pathology, Amsterdam UMC location University of Amsterdam, Amsterdam, The Netherlands; 2 Lymphoma and Myeloma Center Amsterdam (LYMMCARE), Amsterdam, The Netherlands; 3 Cancer Center Amsterdam (CCA), Cancer Biology and Immunology, Target & Therapy Discovery, Amsterdam, The Netherlands; 4 Department of Experimental Immunology, Amsterdam UMC location University of Amsterdam, Amsterdam, The Netherlands; 5 Amsterdam Institute for Infection and Immunity, Cancer Immunology, Amsterdam, The Netherlands; 6 Department of Hematology, Amsterdam UMC location University of Amsterdam, Amsterdam, The Netherlands

**Keywords:** Cancer microenvironment, Apoptosis, Cancer therapeutic resistance, Cell signalling, B-cell lymphoma

## Abstract

Mantle cell lymphoma (MCL), an aggressive, but incurable B-cell lymphoma, is genetically characterized by the t(11;14) translocation, resulting in the overexpression of Cyclin D1. In addition, deregulation of the B-cell lymphoma-2 (BCL-2) family proteins BCL-2, B-cell lymphoma-extra large (BCL-X_L_), and myeloid cell leukemia-1 (MCL-1) is highly common in MCL. This renders these BCL-2 family members attractive targets for therapy; indeed, the BCL-2 inhibitor venetoclax (ABT-199), which already received FDA approval for the treatment of chronic lymphocytic leukemia (CLL) and acute myeloid leukemia (AML), shows promising results in early clinical trials for MCL. However, a significant subset of patients show primary resistance or will develop resistance upon prolonged treatment. Here, we describe the underlying mechanisms of venetoclax resistance in MCL, such as upregulation of BCL-X_L_ or MCL-1, and the recent (clinical) progress in the development of inhibitors for these BCL-2 family members, followed by the transcriptional and (post-)translational (dys)regulation of the BCL-2 family proteins, including the role of the lymphoid organ microenvironment. Based upon these insights, we discuss how rational combinations of venetoclax with other therapies can be exploited to prevent or overcome venetoclax resistance and improve MCL patient outcome.

## Background

Mantle cell lymphoma (MCL) is a rare, but aggressive B-cell lymphoma, defined by the translocation t(11;14), resulting in the constitutive overexpression of cyclin D1 [1]. The disease comprises 3–10% of all non-Hodgkin lymphomas (NHL) and the median age at diagnosis is 60–65 years. MCL is thought to combine the unfavorable features of both indolent and aggressive NHL subtypes, as it is incurable with conventional chemoimmunotherapy and it has a more aggressive disease course [2]. Following relapse upon standard chemoimmunotherapy, with or without autologous transplant, patients are currently being treated with other chemotherapy regimens or with recently developed targeted therapies such as the Bruton’s tyrosine kinase (BTK) inhibitor ibrutinib or the recently approved anti-CD19 chimeric antigen receptor (CAR) T-cell therapy [2]. However, after failure of these salvage therapies, treatment options are strongly reduced.

Targeting apoptosis using the B-cell lymphoma-2 (BCL-2) inhibitor venetoclax is a promising novel therapeutic approach for MCL, with overall response rates (ORR) of 50–75% in early clinical trials, depending upon the number and the type of pretreatments the patients received. However, eventually most patients still relapsed, and complete remission (CR) was only achieved by 18–21% of patients [3–5]. Combining venetoclax with other targeted therapies or chemotherapy regimens might overcome this resistance. Currently, several clinical trials are ongoing in MCL evaluating venetoclax in combination with current standard treatments as lenalidomide or bendamustine (e.g., NCT03295240, NCT03523975). The development of rational combinations based on the underlying mechanisms of resistance are expected to be more efficient and successful.

In this review, based upon insights into the underlying mechanisms of venetoclax resistance, including the role of genetic alterations, transcriptional and (post-)translational regulatory processes, and microenvironment-derived stimuli, we will present potential combination therapies to prevent or overcome venetoclax resistance in MCL.

## Venetoclax resistance

**Fig. 1:**
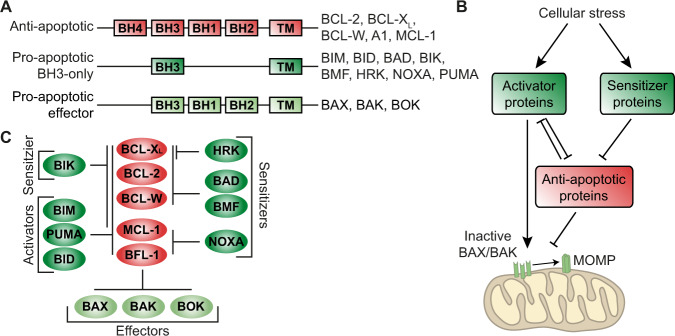
Classification of BCL-2 family proteins and interactions among BCL-2 family proteins. **A** There are three main types of BCL-2 family proteins: anti-apoptotic proteins, pro-apoptotic BH3-only proteins and pro-apoptotic effector proteins. Anti-apoptotic proteins contain all four BH domains, while the pro-apoptotic proteins lack certain domains. Most BCL-2 members also have a transmembrane (TM) domain for anchoring to organelles. **B** Cellular stress induces upregulation of BH3-only proteins. The sensitizer proteins interact with the anti-apoptotic proteins, disrupting their inhibition of BAX/BAK. Activator proteins can also interact with anti-apoptotic proteins, but they can also directly stimulate BAX and BAK to oligomerize and form a pore. **C** Interactions between the subtypes of BCL-2 family proteins. All of the anti-apoptotic proteins can interact with all effectors. Furthermore, they can all interact with the activator BH3-only proteins BIM, PUMA, and Bid. The sensitizer BH3-only proteins have a more selective binding pattern: NOXA only targets MCL-1 and BFL-1, HRK only targets BCL-X_L_, BAD and Bmf are able to antagonize BCL-X_L_, BCL-2, and BCL-W, and Bik interacts with all anti-apoptotic proteins except BFL-1. MOMP mitochondrial outer membrane permeabilization.

Several mechanisms underlying venetoclax resistance have been described, with high levels of the alternative anti-apoptotic proteins myeloid cell leukemia-1 (MCL-1) and/or B-cell lymphoma-extra large (BCL-X_L_) as most outstanding determinants of resistance [6–9]. These anti-apoptotic proteins serve as a buffer for the released pro-apoptotic BH3 proteins, preventing the activation and oligomerization of BAX and BAK (Fig. 1). Elevated MCL-1 or BCL-X_L_ levels can be caused by genetic aberrations and microenvironmental interactions or, in the case of secondary resistance, due to prolonged venetoclax treatment. In MCL cell lines rendered venetoclax-resistant by continuous exposure to venetoclax, upregulation of MCL-1 and, to a lesser extent, of BCL-X_L_ was observed as compared to their sensitive parental counterparts [6, 7, 9]. Notably, comparison of samples from chronic lymphocytic leukemia (CLL) patients before and after venetoclax treatment also revealed upregulation of at least one of the anti-apoptotic proteins by venetoclax treatment [10, 11].

Expression levels of the anti-apoptotic proteins alone is not sufficient to dictate venetoclax sensitivity, the level of occupation of BCL-2 by pro-apoptotic proteins such as BIM also determines the sensitivity to venetoclax. If BCL-2 is occupied by BIM, as is often the case in MCL, BIM can be immediately released upon venetoclax exposure and trigger cell death [7–9]. These cells are so-called primed for death and show better venetoclax responses.

A common mechanism of resistance for other targeted therapies is the acquisition of mutations in (the binding site of) the target of the inhibitor. However, for MCL, only one patient with mutations in the BH3-binding groove of BCL-2 has been reported yet. For CLL, with higher numbers of patients relapsed on venetoclax, *BCL2* mutations have been more frequently observed, mostly after prolonged treatment duration [6, 7, 12–14]. These mutations, e.g., G101V, D103X and V156N, specifically reduce venetoclax binding to BCL-2, while BH3 protein binding was not affected. However, *BCL2* mutations are presented at rather low allele frequencies and it remains unclear whether these infrequent subclones might render the whole malignant population resistant. These mutations are not found in large genomic analyses of biopsies from MCL and CLL patients relapsed from venetoclax plus ibrutinib or venetoclax single-therapy respectively, probably due to too low-resolution sequencing [5, 11, 15, 16]. But these studies did reveal several other genetic aberrations in relapsed patients, involving *CDKN2A/B, CCND1, TP53, NOTCH1/2, ATM, KMT2D* and *SMARCA2/4*, indicating a role for cell cycle regulation and chromatin remodeling. Nonetheless, no pattern was observed in clonal evolution of these resistant clones and the exact role of each individual genetic abnormality in conferring resistance to venetoclax is not clear yet, suggesting that venetoclax resistance is not solely driven by any particular single nucleotide variation, but rather involves complex changes.

In addition to mutational status and involvement of BCL-2 family members, regulators of energy metabolism have been identified as drivers of venetoclax resistance in CLL and diffuse large B-cell lymphoma (DLBCL) [11]. In MCL cells, no such relation between metabolism and venetoclax sensitivity has been reported yet, although metabolites can regulate expression or interactions of BCL-2 family proteins in several cell models, indicating the potential role of energy metabolism in venetoclax resistance [8].

In conclusion, primary venetoclax resistance in MCL is mainly caused by elevated levels of the anti-apoptotic proteins MCL-1 and/or BCL-X_L_ or by decreased priming of BCL-2. In the case of secondary resistance, upregulation of MCL-1 and BCL-X_L_ have also shown to be of great significance, while acquisition of mutations in the target of the inhibitor is rarely observed. Pending mechanistic insight into the role of metabolism, inhibition of MCL-1 and BCL-X_L_, either directly or by targeting upstream regulators, might be most achievable in order to overcome venetoclax resistance in MCL.

## Inhibition of MCL-1 and/or BCL-X_L_

Combining venetoclax with MCL-1 and/or BCL-X_L_ inhibition demonstrated strong synergy in several preclinical models [8, 9]. However, whereas BCL-2 inhibition is already FDA-approved, the search for safe, effective, and selective MCL-1 and BCL-X_L_ inhibitors has proven challenging. In this part, the current status in the development of inhibitors of MCL-1 and BCL-X_L_ will be discussed.

### MCL-1 inhibition

In the past years, several MCL-1-specific BH3-mimetics have entered development, although none of them successfully completed clinical trials yet, due to the key role of MCL-1 in cardiac, neural and hepatic cell survival [6, 7, 17, 18]. Currently, several phase 1 trials are ongoing to evaluate if and how MCL-1 inhibitors can be safely administered to patients. Further progress may involve different (and safer) administration: encapsulation of the MCL-1 inhibitor S63845 in tumor-targeted nanoparticles allowed 3.5-fold reduction in drug dose and more specific drug delivery in a DLBCL xenograft model, minimizing toxicities [19].

### BCL-X_L_ inhibition

The development of BCL-X_L_ inhibitors has been hampered by severe on-target platelet toxicity [6, 9]. Several strategies have been used to mitigate the thrombocytopenia effect of BCL-X_L_ inhibitors, such as the use of proteolysis targeting chimera (PROTAC), antibody-drug conjugates, or prodrugs targeting BCL-X_L_.

PROTACs link a target molecule to a specific E3 ubiquitin ligase, thereby promoting ubiquitination of the target protein. By linking BCL-X_L_ to an E3 ubiquitin ligase which is poorly expressed in platelets, thrombocytopenia might be prevented. Currently, two BCL-X_L_ targeting PROTACs have been reported, XZ424 and DT2216, and both show in vitro potent cytotoxicity against tumor cells while sparing platelets [20]. Recently, DT2216 has entered clinical trials for relapsed and/or refractory solid tumors (NCT04886622).

Apart from PROTACS, the first BCL-X_L_-targeting antibody-drug conjugate, ABBV-155, has entered phase I clinical trials (NCT03595059), even as the pro-drug APG-1252/palcitoclax (NCT03080311, NCT04210037) and the intravenous dendrimer conjugate AZD-0466 (NCT04214093, NCT04865419) [20]. However, no results for these trials have been reported yet and so BCL-X_L_ inhibition is not yet ready for clinical use.

In conclusion, direct inhibition of MCL-1 or BCL-X_L_ to sensitize MCL cells to venetoclax is not yet clinically safe. Therefore, targeting upstream positive regulators of MCL-1 or BCL-X_L_ or negative regulators of pro-apoptotic BH3-only proteins to increase the apoptotic priming might provide a promising alternative. To identify these upstream regulators, the next sections focus on the regulation of BCL-2 family proteins in healthy B cells and on their dysregulation in MCL.

## (Dys)regulation of BCL-2 family proteins in MCL

**Fig. 2:**
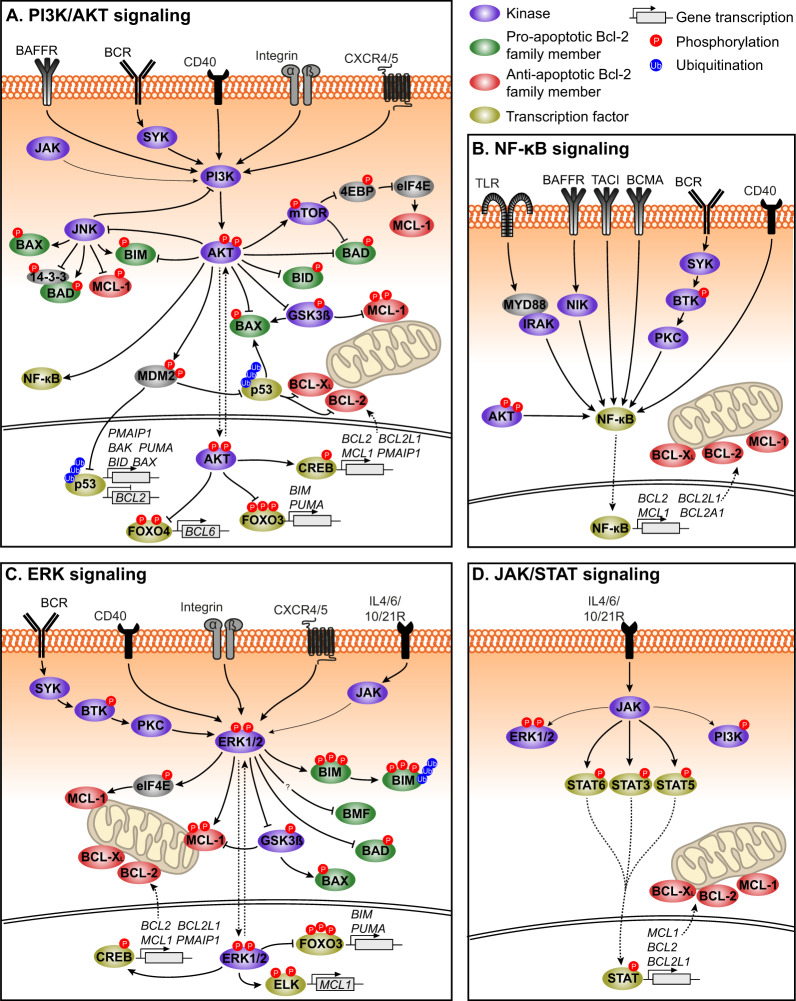
Regulation of BCL-2 family proteins. Stimulation of the BCR, IL-Rs, TLRs, growth factor receptors, integrins, chemokine receptors, or other surface molecules such as CD40 result in downstream activation of **A** PI3K/AKT signaling, **B** NF-κB signaling, **C** ERK signaling, and/or **D** JAK/STAT signaling, all involved in the regulation of BCL-2 family members. Activation of the NF-κB pathway or the JAK/STAT pathway affects the transcription of anti-apoptotic proteins, while activation of the AKT and ERK pathway are also involved in the transcription of pro-apoptotic proteins and in the phosphorylation and the ubiquitination of several BCL-2 family members. 4EBP 4E-binding protein, AKT protein kinase B, BAD BCL-2-associated death promotor, BAFFR BAFF-receptor, BAK BCL-2 homologous antagonist killer, BAX BCL-2-associated X protein, BCL-2 B-cell lymphoma-2, BCL2A1 BCL-2-related protein A1/BFL-1,BCL2L1 BCL-2 like 1, BCL-X_L_ B-cell lymphoma-extra large, BCMA B-cell maturation protein, BCR B-cell receptor, BID BH3-interacting domain death agonist, BIM BCL-2-interacting mediator of cell death, BMF BCL-2-modyfying factor, BTK Bruton’s tyrosine kinase, CD40 cluster of differentiation 40, CREB cAMP response element-binding protein, CXCR4 C-X-C chemokine receptor type 4, eIF4E eukaryotic initiation factor 4E, ELK E-twenty six (ETS) like-1 protein, ERK extracellular signal regulated kinase, FOXO3/4 forkhead box family transcription factors 3/4, GSK3ß glycogen synthase kinase 3ß, IL-4R interleukin 4 receptor, IRAK interleukin-1 receptor associated kinase, JAK Janus kinase, JNK c-Jun N-terminal kinase, MCL-1 myeloid cell leukemia-1, MDM2 mouse double minute 2/E3 ubiquitin-protein ligase, mTOR mammalian target of rapamycin, MYD88 Myeloid differentiation factor 88, NF-κB nuclear factor κB, NIK NF-kappa-B-inducing kinase, PI3K phosphatidylinositol 3-kinase, PKC protein kinase C, PMAIP1 phorbol-12 myristate-13-acetate-induced protein 1/NOXA, PUMA p53-upregulated modulator of apoptosis, STAT signal transducer and activator of transcription, SYK spleen-associated tyrosine kinase, TACI transmembrane activator and CAML [calcium-modulator and cyclophilin ligand] interaction, TLR toll-like receptor.

In healthy B cells the levels of the apoptotic proteins are tightly regulated via several key signaling pathways (Fig. 2). These pathways can be triggered by B-cell receptor (BCR) activation or microenvironmental stimuli and cellular stressors which induce alterations in transcriptional or (post-)translational regulation of the BCL-2 family members. In malignant B cells this regulation is often disturbed, due to genetic abnormalities and/or increased BCR/microenvironmental signaling. In this section, the key regulators of the BCL-2 family members are reviewed, in the context of healthy B cells as well as in the context of MCL.

### Genetic abnormalities

MCL cells harbor genomic lesions mainly leading to BCL-2 overexpression and BIM repression. The major cause of BCL-2 overexpression is the loss of 13q14 (40–60% of cases), which leads to repression of miR-15a and miR-16–1, miRNAs negatively regulating BCL-2 at post-transcriptional level [1, 9, 21, 22]. Furthermore, in ~20% of the cases BCL-2 overexpression is caused by amplification of 18q21, the *BCL2* locus. Lastly, it has been suggested that BCL-2 overexpression may arise from downregulation of F-box only protein 10 (FBXO10), an E3 ubiquitin ligase that targets BCL-2 for proteasomal degradation [23]. The major cause of BIM repression is deletion of *BCL2L11*, the gene encoding BIM, which is observed in 20–30% of patients (Table 1) [1, 6, 21, 22].

**Table 1:** BCL-2 family member dysregulation in MCL.

Protein	Alteration in MCL	Causes
BCL-2	Elevated (~90%) [9, 23, 25]	◦ 13q14.3 loss (40–60%) [9, 21, 22] ◦ 18q21 gain (10–20%) [9, 21, 22] ◦ Reduced *FBXO10* expression [23]
MCL-1	Elevated (30–40%) [9, 25]	◦ Microenvironmental interactions [9, 44]
BCL-X_L_	Elevated (40–60%) [9, 25, 102]	◦ Microenvironmental interactions [9, 32, 44]
BCL-W	Elevated [25, 102]	
BH3-only	BIM reduced [25]	◦ Deletion BIM (20–30%) [6, 21, 22]
P53	Reduced	◦ Loss or mutation (20–40%) [21, 22]

Next to these aberrations, *TP53* dysregulation, either by 17p deletion or *TP53* mutation, is observed in 20–40% of MCL patients [21, 22]. Repression of p53 results in reduced transcription of pro-apoptotic proteins such as BAX and PUMA in response to cellular stress, particularly (chemotherapy-induced) DNA damage, as well as in reduced inhibition of BCL-2 and BCL-X_L_, reduced BAX and BAK activation and reduced BCL-2 transcription suppression (Fig. 2A) [9, 24]. Other genetic alterations often observed in MCL, e.g., those involving SOX11 and cyclin D1, do not affect BCL-2 protein family expression [25].

### Intracellular regulatory pathways

The altered BCL-2 family expression profile in malignant B cells also arises from increased activation of key regulatory pathways of BCL-2 family members compared to healthy B cells (Fig. 2). This is mainly caused by the distinct composition of the lymphoid microenvironment of malignant B cells. Here, first, the key regulatory pathways of BCL-2 family members in B cells in general will be briefly described, followed by the effect of the malignant lymphoid microenvironment on BCL-2 family protein expression in MCL.

#### PI3K/AKT signaling

One of the most prominent apoptosis-regulatory pathways is the phosphatidylinositol 3-kinase (PI3K)/AKT pathway. Activation of AKT induces transcription of anti-apoptotic proteins via amongst others activation of nuclear factor κB (NF-κB) and increases stability and translation of MCL-1 via mammalian target of rapamycin (mTOR) activation and glycogen synthase kinase 3ß (GSK3ß) inhibition, respectively (Fig. 2A) [26–28]. Moreover, AKT represses expression of pro-apoptotic proteins via inhibition of forkhead box family transcription factors (FoxO) and p53 and reduces the activity of BID, BIM, and BAD (Fig. 2A, B) [26, 27]. Lastly, AKT regulates the BCL-2 family proteins via inhibition of the c-Jun N-terminal kinase (JNK) pathway, preventing activation of BAX, BAD and BIM, while priming MCL-1 for phosphorylation by GSK3ß and consequently for degradation. (Fig. 2A) [26, 27].

#### ERK signaling

Activation of ERK also promotes transcription of MCL-1, BCL-2, and BCL-X_L_, and also enhances the translation and the stability of MCL-1, however via phosphorylation of eukaryotic initiation factor 4E (eIF4E) and via direct MCL-1 phosphorylation and GSK3ß repression, respectively (Fig. 2C) [26, 29, 30]. Activated ERK also inhibits pro-apoptotic proteins, by downregulating the transcription of BIM and PUMA and by phosphorylating BIM, BAD and GSK3ß, thereby affecting BAX translation (Fig. 2C) [26, 29, 30].

#### JAK/STAT signaling

Lastly, Janus kinase (JAK)/signal transducer and activator of transcription (STAT) signaling is a major regulator of BCL-2 family proteins. Activation of JAK and the subsequent translocation of STAT induces transcription of MCL-1 and BCL-X_L_ (Fig. 2D) [26, 31]. Activated JAK can also interact with previously mentioned signaling pathways, as it can activate PI3K and ERK signaling [31].

### Microenvironmental regulation of BCL-2 family expression

**Fig. 3:**
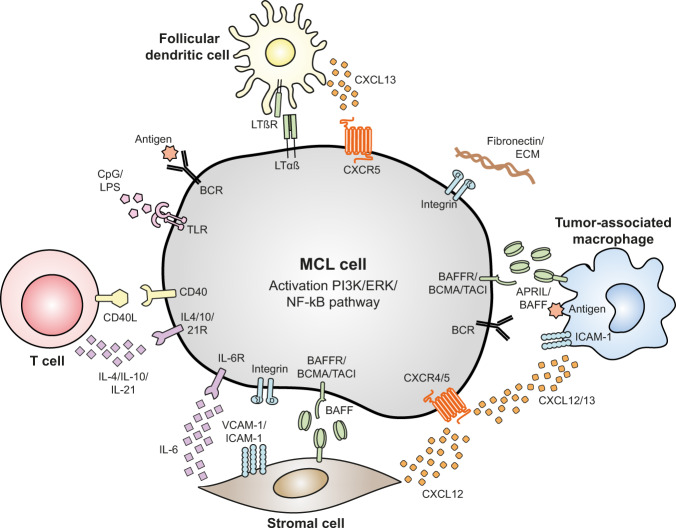
Interactions of mantle cell lymphoma cells in their microenvironment. The different interactions of MCL cells with components from their lymphoid tissues activate major pro-survival pathways such as the PI3K, ERK, and NF-κB pathways. This stimulates the expression and activity of anti-apoptotic proteins, while inhibiting expression and activity of pro-apoptotic proteins. APRIL a proliferation-inducing ligand, BAFF B-cell activating factor, BAFFR BAFF-receptor, BCMA B-cell maturation antigen, BCR B-cell receptor, CD40 cluster of differentiation 4, CD40L CD40 ligand, CXCL12/13 C-X-C motif chemokine ligand-12/13, CXCR4 C-X-C chemokine receptor type 4, ECM extracellular matrix, ERK extracellular signal-regulated kinase, ICAM-1 intracellular cell adhesion molecule-1, IL-6 interleukine-6, IL-6R IL-6 receptor, MCL mantle cell lymphoma, NF-κB nuclear factor κB, LPS lipopolysaccharide, LTαß lymphotoxin αß, LTßR LTß receptor, PI3K phosphatidylinositol 3-kinase, TACI transmembrane activator and CAML [calcium-modulator and cyclophilin ligand] interactor, TLR toll-like receptor, VCAM-1 vascular cell adhesion molecule-1.

The key regulatory pathways of the BCL-2 family members in B cells can be activated by signals from their microenvironmental bystander cells (Fig. 3). The composition of the lymphoid microenvironment of malignant B cells is distinct from that of healthy B cells, thereby affecting the apoptotic balance. In this section, we discuss the effect of the malignant lymphoid microenvironment on BCL-2 family protein expression in MCL.

#### T cells

Mature B-cell malignancies secrete chemokines and cytokines which attract T cells and stimulate T-cell differentiation into T-helper cells and regulatory T cells. These T cells support in turn the growth and survival of the malignant cells via the CD40/CD40L axis, but also via secretion of soluble factors such as IL-4, IL-10, IL-21, and tumor necrosis factor (TNF)-α [32, 33]. CD40 stimulation results in NF-κB signaling, as well as in AKT and ERK signaling, and hence upregulation of especially BCL-X_L_ and MCL-1, although downregulation of pro-apoptotic proteins has also been observed (Fig. 2A–C) [32–34]. The soluble factors primarily activate the JAK/STAT signaling, resulting in transcription of the anti-apoptotic proteins (Fig. 2D).

#### Stromal cells

MCL cells also bi-directionally interact with stromal cells, thereby shifting the expression profile of the stromal cells toward a pro-tumor profile [32]. These stromal cells in turn prevent apoptosis of MCL cells via integrin binding to vascular cell adhesion molecule-1 (VCAM-1) and intracellular cell adhesion molecule-1 (ICAM-1), via secretion of chemokines such as C-X-C motif chemokine ligand-12 (CXCL12) and -13 and via expression of B-cell activating factor (BAFF). This results in activation of AKT, ERK and NF-κB signaling (Fig. 2A–C), and thus in upregulation of MCL-1, BCL-X_L_ and BCL-2 [32, 33, 35, 36]. Interestingly, direct adhesion to stromal cells was critical for their full protective effect [36].

#### Macrophages

The third group of cells which modulate the survival of lymphoma cells are the macrophages. MCL cells attract monocytes and promote them to differentiate into tumor-associated M2 macrophages (TAM) [37, 38]. The precise role for TAMs in MCL is still unknown, although recent studies indicate that TAMs induce MCL growth via secretion of, e.g., IL-10 and BAFF [32, 37, 38]. Interestingly, T cells also play a role by CD40-mediated induction of IL-32β, which in turn instructs the TAMS to secrete BAFF [38, 39]. This is in concordance with the more established role of TAMs in CLL, where TAMs induce lymphoma survival via secretion of a proliferation-inducing ligand (APRIL), BAFF, CXCL12 and -13, and Wnt5a, and via stimulation of the BCR and CD38 [40, 41]. These stimuli activate NF-κB, AKT and ERK, resulting in upregulation of anti-apoptotic proteins, such as BCL-2, BCL-X_L_, and BFL-1 and downregulation of BAD (Fig. 2A–C) [28, 42].

#### Lymphoma cells

Apoptotic tumor cells themselves can also produce signaling molecules that affect apoptosis of their neighboring tumor cells. For example, it has recently been described that apoptotic stress caused by BH3-mimetics in HeLa cells induces fibroblast growth factor (FGF)-2 secretion, which leads to ERK-dependent transcriptional upregulation of pro-survival BCL-2 proteins in the neighboring cells [43]. Whether apoptotic MCL cells also secrete such paracrine survival factors upon stress, remains to be determined.

#### Other microenvironmental factors

The lymphoid microenvironment further consists of non-cellular components which affect the apoptotic priming of MCL cells, such as extracellular matrix (ECM), antigens and bacterial epitopes. Binding of malignant B cells to ECM components activates the AKT, ERK, and NF-κB pathways and the concomitant shift in apoptotic priming [32]. Antigens, either bound to TAMs or stromal cells or in suspension, activate the BCR and thereby primarily elevate MCL-1 levels via the AKT pathway and BCL-X_L_ via the NF-κB pathway (Fig. 2A, B) [32, 44, 45]. Lastly, microbial epitopes present in the LN, such as CpG and lipopolysaccharides (LPS), activate toll-like receptors (TLRs), resulting in stimulation of amongst others the NF-κB pathway and thus expression of BCL-X_L_ (Fig. 2B) [34, 45, 46].

Taken together, MCL cells show dysregulation of BCL-2 family members, with elevated levels of the anti-apoptotic proteins, either due to genetic aberrations (BCL-2) or microenvironmental stimuli (MCL-1 and BCL-X_L_), and with decreased levels of pro-apoptotic proteins, mostly caused by genetic aberrations (Table 1). The microenvironmental effect is supported by gene expression studies, showing upregulation of BCR and NF-κB pathway target genes specifically in MCL cells in the lymph node as compared to peripheral blood [44].

## Strategies to overcome primary and secondary venetoclax resistance

Targeting key signaling cascades that engage molecules such as AKT or ERK or disruption of microenvironmental interactions might synergize with venetoclax to induce cell death of the MCL cells, while sparing the platelets and cardiac cells. In this part, we will discuss the most promising strategies to overcome venetoclax resistance.

### Inhibition of the BCR signalosome

**Fig. 4:**
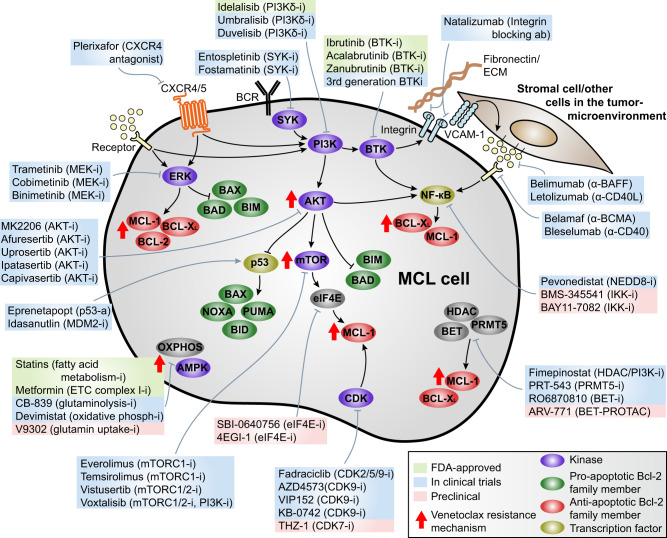
Rationale drug combinations to enhance venetoclax sensitivity in MCL cells. Targeting the venetoclax resistance mechanisms, depicted with a red arrow, and their regulators, provides opportunities to sensitize MCL cells to venetoclax. Drugs in green boxes are FDA-approved for MCL, drugs in blue boxes are currently evaluated in clinical trials, and drugs in red boxes have only been tested preclinically. The i in the boxes indicates an inhibitor, the a an activator. The ‘receptor’ indicates an universal receptor, reflecting growth factor receptors, toll-like receptors, cytokine receptors, etc. For more details regarding the receptor, see Figs. 2 and 3. ab antibody, AKT protein kinase B, AMPK AMP (adenosine monophosphate)-activated protein kinase, BAD BCL-2-associated death promotor, BAFF B-cell activating factor, BAX BCL-2-associated X protein, BCMA B-cell maturation antigen, BCL-2 B-cell lymphoma-2, BCL-X_L_ B-cell lymphoma-extra large, BCR B-cell receptor, BET bromodomain and extra-terminal domain, BID BH3-interacting domain death agonist, BIM BCL-2-interacting mediator of cell death, BTK Bruton’s tyrosine kinase, CDK cyclin-dependent kinase, CXCR4 C-X-C chemokine receptor type 4, eIF4E eukaryotic initiation factor 4E, ERK extracellular signal regulated kinase, ETC electron transport chain, HDAC histone deacetylase, IKK IκB kinase, MCL-1 myeloid cell leukemia-1, MDM2 mouse double minute 2/E3 ubiquitin-protein ligase, MEK mitogen-activated protein kinase kinase, mTOR mammalian target of rapamycin, mTORC1/2 mTOR complex 1/2, NEDD8 neural precursor cell expressed developmentally downregulated protein 8, NF-κB nuclear factor κB, OXPHOS oxidative phosphorylation, PI3K phosphatidylinositol 3-kinase, PRMT5 protein arginine methyltransferase 5, PROTAC proteolysis targeting chimera, PUMA p53-upregulated modulator of apoptosis, SYK spleen-associated tyrosine kinase.

Suppression of integrin activation by inhibition of kinases from the BCR signalosome, such as BTK and PI3Kδ, mobilizes MCL and CLL cells from the LN to PB, disrupting the growth- and survival-supportive microenvironmental interactions (Fig. 4) [47, 48]. Mobilized CLL cells obtained from the PB of patients after treatment with the BTK-inhibitor ibrutinib showed reduced MCL-1 and BCL-X_L_ levels and enhanced BIM levels compared to pre-ibrutinib samples [10, 49]. It is tempting to speculate that similar effects will be accomplished upon ibrutinib-evoked mobilization of MCL cells.

An additional advantage of inhibiting the BCR signalosome is the reduced activity of the downstream NF-κB and AKT pathways and the consequent effects on the regulation of several BCL-2 family members (Fig. 4) [49–51]. Moreover, these inhibitors may also directly target the microenvironment: in CLL patients, T-cell activation and proliferation is strongly reduced after inhibition of the BCR signalosome, thereby preventing T-cell-mediated upregulation of MCL-1 and BCL-X_L_ in CLL cells and subsequent venetoclax resistance [52].

Currently, several clinical trials assessing the effectiveness of the combination of venetoclax and ibrutinib are ongoing, and thus far, they show impressive results (Table 2). The AIM study, a phase II study of ibrutinib and venetoclax in 24 patients with R/R MCL, demonstrated an ORR of 71% and a CR of 63% at 16 weeks of treatment [53]. Another phase II study with additional obinutuzumab treatment included, shows even a small beneficial effect over the AIM study, however, longer follow-up will show if this translates into prolonged remission duration [54]. Treatment with these drug combinations have also demonstrated high response rates in CLL and the first results of the phase III trial of venetoclax and ibrutinib are also promising, firmly establishing the combination of ibrutinib and venetoclax as therapeutic option in MCL [55–57].

**Table 2:** Clinical trials with venetoclax in MCL.

Interventions	Conditions	Phase	NCT Identifier	Start date
Ven + **Ibrutinib**	R/R MCL	I	NCT02419560	Apr 2015
Ven + **Ibrutinib** (AIM) [53]	R/R MCL	II	NCT02471391	Jun 2015
Ven + **Ibrutinib**	R/R MCL	II	NCT04477486	Sep 2020
Ven + **Ibrutinib** (SYMPATICO) [55]	TN & R/R MCL	III	NCT03112174	Jun 2017
Ven + **Acalabrutinib**	R/R MCL	II	NCT03946878	Aug 2019
Ven + *Rituximab*	TN MCL	II	NCT05025423	Jun 2022
Ven + **Ibrutinib** + *Obinutuzumab* (OASIS) [54]	R/R MCL	I/II	NCT02558816	Oct 2015
Ven + **Ibrutinib** + *a-CD20* (OASIS-II)	TN MCL	II	NCT04802590	Jan 2022
Ven + **Pirtobrutinib** + *Rituximab* (BRUIN)	R/R MCL/CLL/NHL	I/II	NCT03740529	Nov 2018
Ven + **Zanubrutinib** + *Obinutuzumab* (BOVen) [96]	TN *TP53*-mut MCL/CLL	II	NCT03824483	Feb 2019
Ven + **Acalabrutinib** + *Obinutuzumab*	TN & R/R MCL	I/II	NCT04855695	Jul 2021
Ven + **Ibrutinib** + Chemoimmunotherapy (WINDOW-2) [97]	TN MCL	II	NCT03710772	May 2019
Ven + **Ibrutinib** + Lenalidomide + *Obinutuzumab* + Prednisone (VIPOR) [98]	TN & R/R MCL	I/II	NCT03223610	Feb 2018
Ven + Lenalidomide + *Rituximab* [99]	TN MCL	I	NCT03523975	Dec 2018
Ven + Lenalidomide + *Rituximab* (VALERIA)	R/R MCL	I/II	NCT03505944	Jul 2018
Ven + Bendamustine + *Rituximab*	TN MCL	II	NCT03834688	Jan 2020
Ven + Bendamustine + *Obinutuzumab*	TN MCL	II	NCT03872180	Apr 2019
Ven + Bendamustine + *Rituximab* + **Ibrutinib**	R/R MCL	I	NCT03295240	Sep 2017
(Ven or Bendamustine) + *Rituximab* + **Acalabrutinib** [95]	TN MCL	I	NCT02717624	May 2016
Ven + Bendamustine + *Rituximab* + Cytarabine	TN high-risk MCL	II	NCT03567876	Sep 2018
(Ven + *Rituximab* or Bendamustine) + APR-246	R/R *TP53*-mut MCL/CLL/RT	I/II	NCT04419389	Mar 2021
Ven + Polatuzumab Vedotin + *Rituximab*/Hyaluronidase	R/R MCL	II	NCT04659044	Apr 2021
Ven + Copanlisib	R/R MCL	I/II	NCT04939272	Jun 2022
(Ven or Lenalidomide) + *Ublituximab* + Umbralisib	R/R MCL/CLL/NHL	I/II	NCT03379051	Mar 2018

Drugs in bold target BTK, drugs in italic target CD20, drugs in underline are current standard chemoimmunotherapy regimens.

*CLL* chronic lymphocytic leukemia, *MCL* mantle cell lymphoma, *NHL* non-Hodgkin lymphoma, *R/R* refractory/relapsed, *RT* richter transformation, *TN* treatment-naive, *TP53-mut* TP53 mutant, *Ven* venetoclax.

### Inhibition of integrin activation

Apart from inhibition of the BCR signaling, CXCR4 antagonists (e.g., plerixafor/AMD3100) or integrin-blocking antibodies (e.g., natalizumab) disrupt microenvironmental interactions and hereby also mobilize lymphoma cells (Fig. 4) [36, 58, 59]. Furthermore, we have recently demonstrated that targeting of hematopoietic cell kinase (HCK) also impairs adhesion of MCL cells to the ECM and stromal cells [60]. Natalizumab and plerixafor are well tolerated in early clinical trials [59], for HCK inhibitors, no clinical trials have been reported yet.

### Inhibition of AKT signaling

Inhibition of the PI3K/AKT pathway is associated with the reduction of especially MCL-1 levels, but also with reduction of BCL-X_L_ levels and accumulation of BAD and BIM (Fig. 4) [61, 62]. Moreover, both intrinsic and acquired venetoclax resistance have been associated with enhanced AKT activation, and concomitant susceptibility to PI3K/AKT inhibition [62–64]. Therefore, synergism between PI3K/AKT/mTOR inhibitors and venetoclax have frequently been investigated and observed in vitro [8, 33, 61–64]. Whereas clinical development of AKT and mTOR inhibitors have been hampered due to toxicities and limited efficacy, several clinical trials with PI3K inhibitors are ongoing. For MCL, no results have been reported yet for the trials combining venetoclax with PI3K inhibitors (NCT03379051, NCT04939272; Table 2), but for R/R CLL early results are encouraging, with ORRs of 89% (8/9) and 85% (11/13) for venetoclax combined with either duvelisib (PI3Kδ/γ inhibitor) or with umbralisib (PI3Kδ inhibitor) and ublituximab (anti-CD20) respectively. Although high rates of neutropenia and thrombocytopenia were observed, infections were infrequent, and phase II trials are currently ongoing [65, 66].

### Inhibition of protein translation

The synergy between AKT inhibition and venetoclax can partly be explained by mTOR-mediated reduction in translation of amongst others MCL-1 (Fig. 4). To prevent AKT-mediated toxicities of a combination therapy, inhibition of translation itself might also be a good approach to potentiate venetoclax activity in lymphoma cells. Indeed, ribosomal inhibition using homoharringtonine (HHT) or disruption of the interaction between eIF4E and eIF4G using SBI-0640756 or 4EGI-1 reduces MCL-1 and BCL-X_L_ levels in MCL cell lines and primary CLL cells and potentiated the activity of BH3-mimetics [67–69]. Furthermore, we have recently established that targeting casein kinase 2 (CK2) in MCL cell lines and primary MCL samples represses MCL-1 translation and thereby synergizes with venetoclax (Thus et al. submitted). Clinical data in which venetoclax is combined with disruption of translation are currently lacking.

### Inhibition of ERK signaling

Inhibition of the ERK pathway in combination with venetoclax is also an interesting option, since this pathway is a major regulator of BCL-2 family proteins as well (Fig. 2C). Whereas for AML the combination of venetoclax with ERK pathway inhibitors has frequently been reported and is currently evaluated in clinical trials, for mature B-cell lymphomas this combination is poorly studied. Still, the few reports in which this combination is studied do report synergy in CLL, multiple myeloma (MM), and DLBCL [70–72]. However, this is not observed in MCL: whereas the MEK1/2 inhibitor trametinib synergized with venetoclax in most CLL and MM cell lines and primary CLL samples, this only occurred in two out of seven MCL cell lines [72]. Thus this combination may hold promise for clinical development in CLL and MM, but most likely not for MCL.

### Inhibition of NF-κB activation

Since NF-κB signaling has a central role in the transcription of the anti-apoptotic BCL-2 family proteins (Fig. 2B), combining venetoclax with inhibition of NF-κB signaling might also be a promising approach to increase the efficacy of venetoclax in patients. Indeed, prevention of IκB degradation in MCL cell lines and primary samples downregulated BCL-X_L_ and in some cases also MCL-1 or BCL-2 [34, 41, 73]. Furthermore, inhibition of the non-canonical NF-κB pathway using an NF-κB inducing kinase (NIK) inhibitor in MCL and CLL reduced BCL-X_L_ levels and led to increased vulnerability to venetoclax [38, 74]. These results demonstrate the opportunities for such combination strategies, however, whereas several NF-κB inhibitors have been developed over the years, none of them have entered clinical practice yet, due to severe toxicities [75].

Recently, a phase II clinical trial has been launched in which NF-κB signaling is indirectly targeted in R/R CLL, the BeliVeR trial (NCT05069051). This triple combination of belimumab, a BAFF-neutralizing antibody which is FDA-approved for systemic lupus erythematosus (SLE), venetoclax and rituximab prevented BAFF-induced venetoclax resistance in CLL in vitro [76]. If indeed beneficial for CLL patients, it would also be worthwhile to evaluate this for MCL. In addition, it would be worthwhile to evaluate BCMA-targeted antibody-drug conjugates (ADCs) in combination with venetoclax, as BCMA is one of the receptors for BAFF (Fig. 3) and is expressed on primary MCL in the LN [77]. The safety and tolerability of several of these ADCs are already under evaluation in clinical trials [78].

### Inhibition of CDKs

Inhibition of cyclin-dependent kinases (CDKs) also provides a potential strategy to reduce MCL-1 levels and thereby increase venetoclax sensitivity in lymphoma cells. Several CDKs regulate MCL-1 levels, such as CDK2, which phosphorylates MCL-1, preventing its ubiquitination and binding to BIM, and CDK7 and CDK9, which are involved in the transcriptional regulation of MCL-1 by interacting with RNA polymerase II [79]. Due to the short half-life of MCL-1, MCL-1 is particularly susceptible to disruption of transcriptional activity. Moreover, venetoclax-resistant MCL cells show transcriptional remodeling and thereby increased susceptibility to for example CDK7 inhibition, emphasizing the potential of combining venetoclax with CDK inhibitors (Fig. 4) [5, 16, 80].

Although the rationale behind combining CDK inhibitors with venetoclax is strong and the combination synergizes in vitro, pan-CDK inhibitors such as alvocidib/flavopiridol and dinaciclib show low levels of clinical activity and/or have been plagued with toxicity in vivo [79]. To circumvent this toxicity, more specific and structurally different inhibitors have recently been developed, which induce MCL-1 reduction and sensitize cells to venetoclax in preclinical models [79–82]. For example, fadraciclib, a CDK2/9 inhibitor, completed a phase II trial in solid cancer patients without severe toxicities and is currently tested in R/R CLL in a phase I trial combined with venetoclax (NCT03739554) [82]. Thus, sensitizing MCL cells to venetoclax by using CDK inhibitors might be effective when safe CDK inhibitors have been designed.

### Epigenetic regulation

Another approach to sensitize MCL cells to venetoclax is by targeting the expression of BCL-2 family proteins using epigenetic inhibitors. Inhibition of bromodomain extra-terminal (BET) proteins, histone deacetylases (HDACs) or protein arginine methyltransferases (PRMT5) affect expression of BCL-X_L_, MCL-1 and BIM and synergize in vitro and in MCL mouse models with venetoclax (Fig. 4) [83–86]. Phase I clinical trials of the combination of venetoclax and the BET inhibitor R06870810 or the dual HDAC and PI3K inhibitor fimepinostat (CUDC-907) in R/R DLBCL showed manageable safety profiles and durable anti-tumor activities [87, 88], but in MCL no clinical trials have been initiated yet.

### Inhibition of metabolism

Since regulators of energy metabolism have also been established as drivers of venetoclax resistance and since cells which progress on venetoclax treatment show increased oxidative phosphorylation and AMPK signaling compared to before progression, targeting the metabolism might also potentiate venetoclax activity in MCL cells [8, 11]. Indeed, inhibition of glutamine uptake and its downstream pathways, the AMPK pathway or the electron transport chain, all overcome venetoclax resistance in several MCL cell lines and primary CLL samples [11, 89–91]. Moreover, in retrospective analyses of three clinical studies of CLL, background statin use was associated with an enhanced number of complete responses to venetoclax, although this was ascribed to upregulation of PUMA rather than to a metabolic pathway [90]. Despite the potential of combining inhibition of metabolism with venetoclax, no such clinical trials have been launched for MCL. For CLL, a phase 1 trial that combines venetoclax with the potent HMG-CoA reductase inhibitor pitavastatin has recently been initiated (NCT04512105). If results are promising, it might be worthwhile to extend this trial to MCL.

## Conclusion and future directions

**Fig. 5:**
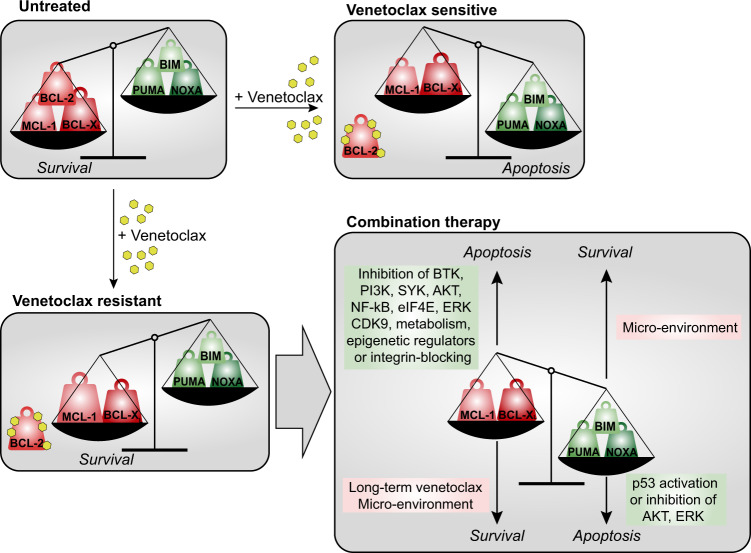
Tipping the balance of MCL toward venetoclax sensitivity by rational combination therapies. In the basal, untreated situation, the apoptotic balance is tilted toward anti-apoptotic proteins (upper left panel). Venetoclax treatment removes BCL-2 from the balance, resulting in apoptosis in the sensitive cells (upper right panel), but the balance is still tilted toward anti-apoptotic proteins in resistant cells, due to for example enhanced MCL-1 or BCL-X_L_ levels (lower left panel). Combining venetoclax with other targeted therapies that may either decrease the amount of MCL-1 or BCL-X_L_ or increase the levels of the pro-apoptotic proteins will tip the balance toward apoptosis. Long-term venetoclax treatment or microenvironmental stimulation counteract these effects by increasing the anti-apoptotic effects and decreasing the pro-apoptotic effects (lower right panel).

To increase the efficacy of venetoclax therapy in MCL, combining venetoclax with inhibitors targeting the other anti-apoptotic BCL-2 proteins MCL-1 and BCL-X_L_ would be an excellent strategy. Although active development of MCL-1 and BCL-X_L_ inhibitors is ongoing, it is uncertain if these inhibitors will maintain a sufficient safety profile for widespread use. Therefore, indirect targeting of the expression of these proteins could be an attractive alternative, for example by the use of BCR signalosome or NF-κB inhibitors (Fig. 5).

Of the various strategies to sensitize MCL cells to venetoclax as discussed in this review, the most advanced strategy is venetoclax combined with BCR signalosome inhibitors, specifically BTK and PI3K inhibitors. These inhibitors efficiently reduce expression of MCL-1 and BCL-X_L_ without causing severe toxicities such as thrombocytopenia, as they specifically target B cells. The other rational combinations discussed show promising preclinical results, however most of them are not (yet) clinically approved.

With only a few targeted therapies being clinically approved yet, venetoclax is currently extensively evaluated in combination with therapies with a less strong mechanistic rationale, such as anti-CD20 therapy and (targeted) chemotherapy (Table 2). Anti-CD20 therapy, e.g., rituximab and obinutuzumab, showed impressive results in CLL in clinical trials and is recently FDA-approved for CLL [92, 93]. The precise underlying mechanism is unknown, although anti-CD20 antibodies have been shown to counteract CD40-induced resistance in CLL cells in vitro irrespective of BCL-2 family member alterations [94]. In MCL, venetoclax and anti-CD20 therapy is often being evaluated in combination with chemotherapeutics or with targeted therapies such as BTK inhibition (Table 2). As in CLL, early results show high response rates and good tolerability in various MCL patient groups, although follow-up time is short [95–99]. To reduce side-effects of standard chemotherapeutics, a phase II trial which combines venetoclax with rituximab and a targeted chemotherapeutic, polatuzumab vedotin, has been launched (NCT04659044; Table 2).

Other drugs which are interesting to evaluate in combination with venetoclax are the multi-kinase inhibitors sorafenib and sunitinib. These inhibitors are already FDA-approved for solid tumors and have been shown to reduce MCL-1 levels in CLL cells, and in the case of sunitinib also BCL-X_L_ and BFL-1 levels [100, 101]. Sunitinib was identified in a screen with 320 kinase inhibitors as most effective synergizer with venetoclax and can also partially overcome CD40L-induced venetoclax resistance, highlighting the opportunities of this drug combination [101].

Which of the rational combinations will be most successful, will also vary per patient. Ideally, personalized drug combinations will be established using drug combination screens ex vivo, or predictive biomarkers or mutation profiles can be used, however, this is not feasible yet. To gain insight into which combination of drugs is best for a specific patient, more clinical trials and more studies into potential biomarkers are needed.
